# Patient satisfaction with the implementation of the family medicine model at Nassem Health Centre, Kuwait

**DOI:** 10.3389/fmed.2026.1873673

**Published:** 2026-07-23

**Authors:** Lulua Alasousi, Abdullah M. Al-Harran, Wafa Al-Rasheedi, Abdulaziz Thawini

**Affiliations:** 1 Ministry of Health of Kuwait, Kuwait City, Kuwait; 2 College of Medicine & Medical Sciences, Arabian Gulf University, Manama, Bahrain

**Keywords:** family practice, patient outcome assessment, patient satisfaction, physicians, primary health care, referral and consultation, surveys and questionnaires

## Abstract

**Background and objectives:**

Primary health care centres (PHCCs) are the foundation stone of Kuwait’s public healthcare system, providing accessible and comprehensive services. Patient satisfaction is a critical indicator of healthcare quality and is associated with improved adherence to medical advice and better health outcomes. Despite international evidence supporting structured family medicine and appointment-based systems, Kuwait’s PHCCs continue to operate primarily on a walk-in basis. This study aimed to describe patient satisfaction with a family medicine appointment clinic at the Nassem PHCC in Kuwait.

**Methods:**

A cross-sectional observational study was conducted between September 2024 and September 2025. The extended recruitment period was necessary to achieve an sufficient sample and capture routine service-utilisation patterns across a full annual cycle. Consecutive patients attending the family medicine appointment clinic were recruited using convenience sampling. A validated, self-administered seven-item questionnaire assessed satisfaction with the doctor–patient relationship using a five-point Likert scale (1 = Very Dissatisfied to 5 = Very Satisfied). After excluding 11 incomplete records from 264 initial responses, the final analytic sample comprised 253 participants. All analyses were conducted in Jamovi (version 2.5).

**Results:**

Principal component analysis confirmed a unidimensional scale structure (KMO = 0.949; Bartlett’s *χ*^2^ = 2,210, *df* = 21, *p* < 0.001). Internal consistency was excellent (Cronbach’s *α* = 0.969). Item means ranged from 4.67 to 4.76, with 74.0–81.0% of respondents selecting the highest satisfaction category across all items, indicating a pronounced ceiling effect. The composite satisfaction score was obviously negatively skewed (skewness = −3.59, kurtosis = 18.0). A statistically significant age-group difference in satisfaction was observed (Kruskal–Wallis *χ*^2^ = 24.60, *df* = 4, *p* < 0.001, *ε*^2^ = 0.098); no significant differences were found by nationality or gender.

**Conclusion:**

Patients attending the Nassem PHCC family medicine appointment clinic reported high Overall Satisfaction. A significant age-related variation was observed, with older patients reporting higher satisfaction than younger adults. Ceiling effects, cross-sectional design, convenience sampling, and potential social desirability bias limit the conclusions that can be drawn. Future research should employ longitudinal designs, comparator groups, and more sensitive instruments.

## 1 Introduction

Primary health care centres (PHCCs) are the foundation of Kuwait’s public healthcare system, providing preventive, acute, and chronic disease services to the population. As the first point of contact for most patients, PHCCs play a central role in ensuring accessible, high-quality healthcare. Patient satisfaction is a widely recognised indicator of healthcare quality and is associated with improved treatment adherence, healthcare utilisation, and patient outcomes (1). As the first point of contact for most patients, PHCCs play a vital role in ensuring equitable access to care and maintaining the overall health of the population. Therefore, evaluating the quality of services delivered at these centres is essential.

Among the various indicators of healthcare quality, patient satisfaction and continuity of care are considered two of the most important measures. High levels of patient satisfaction are associated with improved adherence to medical advice, stronger patient loyalty, and better health outcomes.

Continuity of care, a core principle of family medicine, promotes an ongoing therapeutic relationship between patients and their physicians and has been associated with improved patient experiences and health outcomes (2, 3). Together, these dimensions are widely recognized as essential components of high-quality healthcare systems, as they foster trust, improve outcomes, and strengthen the patient–provider relationship (4).

Patient-centered care theory posits that healthcare quality improves when providers develop a deeper understanding of patients’ preferences, values, and needs. Continuity of care facilitates the development of this understanding over time, leading to more individualized care, stronger therapeutic relationships, and increased patient satisfaction (5). Furthermore, a scoping review of studies conducted across Gulf Cooperation Council (GCC) countries found that patient satisfaction with primary care services is generally high and is influenced by factors such as communication, accessibility, continuity of care, and overall service quality (6).

In many developed healthcare systems, family medicine services within primary care settings are supported by structured appointment systems (2). This organizational approach offers several advantages. Appointment systems enable healthcare providers to organize patient flow more effectively and plan daily clinical activities efficiently (7, 8). They also enhance accessibility by reducing overcrowding and balancing patient demand with available resources. Evidence indicates that appointment systems positively influence patient satisfaction by minimizing waiting times, improving service organization, and creating a more efficient and patient-centered healthcare environment (8). For instance, a 2023 study conducted in Saudi Arabia evaluating the “Mawid” electronic appointment system reported high levels of patient satisfaction, attributed to reduced waiting times, improved access to healthcare services, and better patient flow management (9).

Regional evidence further highlights the effectiveness of appointment systems in primary care settings. Studies from neighboring countries have demonstrated strong patient support for mixed models that combine scheduled appointments with walk-in services (10). Such hybrid systems provide flexibility for patients requiring immediate access while maintaining the efficiency of structured scheduling. Evidence suggests that these approaches can reduce waiting times, improve service delivery, and enhance overall patient satisfaction (11). Surveys in the region consistently show a preference for appointment-based systems, reflecting the growing demand for more organized and accessible primary care services (9).

Despite Kuwait’s substantial investment in healthcare—higher than many other Gulf countries, particularly in infrastructure and pharmaceuticals—its primary healthcare system continues to face several challenges, including long waiting times and clinic overcrowding (12). A key contributing factor is that Primary Health Care Centers (PHCCs) predominantly operate on a walk-in basis, where patients are seen on a first-come, first-served basis. Appointment-based services remain limited and are primarily restricted to specific clinics, such as chronic disease services (13). This reliance on walk-in systems has led to operational inefficiencies, including fragmented service delivery, poor patient flow management, and reduced patient satisfaction. These challenges compromise the efficiency and effectiveness of PHCCs and limit their ability to provide timely, coordinated, and patient-centered care and physician burnout (14). Consequently, there is a growing need to implement structured appointment systems and improve service organization to enhance the quality and accessibility of primary healthcare services in Kuwait.

Against this background, the present study aims to describe patient satisfaction with a family medicine appointment clinic at Nassem PHCC in Kuwait City, evaluate the psychometric properties of the satisfaction instrument, including its internal consistency and factor structure, and examine whether satisfaction varied according to age, gender, or nationality. Given the cross-sectional design and absence of a comparator group, the study was intended to describe patient perceptions rather than establish causal relationships.

## 2 Methods

### 2.1 Study design

This was a cross-sectional observational study conducted between September 2024 and September 2025 at the Nassem PHCC in Kuwait City, Kuwait. A cross-sectional design was selected as appropriate for measuring the prevalence and distribution of patient satisfaction at a single point in time, consistent with standard approaches in health services research (15).

The study was conducted at the Nassem PHCC, where a dedicated family medicine appointment clinic operates on a weekly basis. A team of family medicine specialists is assigned to specific days, and patients are scheduled to follow up with the same physician at each visit to ensure continuity of care. Each consultation is allocated a 20-min time slot.

The target population comprised patients who attended the family medicine appointment clinic during the study period. Patients were excluded if they attended specifically for chronic disease clinic appointments, or declined to provide electronic informed consent. The initial dataset comprised 264 responses; after excluding 11 incomplete records using complete-case analysis, the final analytic sample consisted of 253 participants.

Non-probability convenience sampling was employed. Participating physicians invited all eligible patients attending the clinic on days when a research-designated physician was present.

Eligible participants were directed to a secure online platform via a QR code or hyperlink provided by their physician. The first page of the questionnaire contained an electronic informed consent form; only participants who provided consent proceeded to complete the survey. Participation was voluntary and anonymous, and completion of the survey did not affect the care received.

### 2.2 Questionnaire

A validated questionnaire adapted from Baker (16) was used to assess satisfaction with the doctor–patient consultation. The instrument was originally developed and validated in English for general practice settings in the United Kingdom. For this study, the questionnaire was translated into Arabic independently by two bilingual family medicine specialists. Discrepancies between the two translations were resolved by consensus. A back-translation from Arabic to English was subsequently performed by a third bilingual specialist to verify conceptual equivalence and ensure that no items had acquired unintended meaning during translation. The Arabic version was then reviewed by a consultant in family medicine with expertise in Kuwaiti primary care to evaluate cultural relevance, linguistic clarity, and item appropriateness within the local healthcare context; minor idiomatic adjustments were made following this review.

Responses were recorded on a five-point Likert scale coded as follows: 1 = Very Dissatisfied, 2 = Dissatisfied, 3 = Unsure, 4 = Satisfied, and 5 = Very Satisfied. Higher scores therefore indicate greater satisfaction.

### 2.3 Ethical considerations

Ethical approval was obtained from the Ministry of Health of Kuwait (approval no. 2024/2617). All participants provided electronic informed consent prior to completing the survey. Participation was voluntary, anonymous, and did not affect clinical care.

## 3 Statistical analysis

All statistical analyses were conducted using Jamovi (17) (version 2.5). The significance threshold was set at *α* = 0.05 for all tests, and all *p*-values were two-sided.

Descriptive statistics (frequencies, percentages, means, and standard deviations) were computed for demographic and item-level variables.

Construct validity was evaluated using principal component analysis (PCA). Prior to extraction, the suitability of the correlation matrix was confirmed using the Kaiser–Meyer–Olkin (KMO) measure of sampling adequacy and Bartlett’s test of sphericity (18). The number of components to retain was determined by inspection of the scree plot and the eigenvalue-greater-than-one criterion (19).

The internal consistency of the seven-item scale was assessed using Cronbach’s alpha (*α*). Item-rest correlations (the correlation between each item and the total scale score excluding that item) and alpha-if-item-deleted values were computed to evaluate the contribution of each item to overall reliability (20).

Distributional properties of the composite Overall Satisfaction score (the mean of the seven items) were assessed by computing skewness and kurtosis with their standard errors. Marked departure from normality (indicated by large absolute skewness and kurtosis values) informed the selection of non-parametric inferential tests.

Group differences in the composite Overall Satisfaction score across demographic subgroups were examined using the Kruskal–Wallis one-way analysis of variance by ranks (21). Effect sizes were reported as epsilon-squared (*ε*^2^), interpreted following guidelines by Tomczak small ≈ 0.01, medium ≈ 0.06, large ≈ 0.14. Pairwise *post-hoc* comparisons were conducted using Dunn’s test with Bonferroni correction to control the familywise error rate (22). A *post-hoc* power analysis was conducted to estimate achieved statistical power for the primary comparison.

## 4 Results

### 4.1 Sample characteristics

The initial dataset comprised 264 responses. After excluding 11 incomplete records, the final analytic sample consisted of 253 participants (Table 1). The largest age group was 46–65 years (*n* = 103, 40.7%), followed by 25–45 years (*n* = 92, 36.4%). Participants aged under 18 years accounted for 3.2% (*n* = 8). Most participants were Kuwaiti nationals (*n* = 206, 81.4%) and female (*n* = 138, 54.5%).

**Table 1:** Sample characteristics.

Characteristic	*N* = 253
Age	
<18 years	8 (3.2%)
18–24 years	20 (7.9%)
25–45 years	92 (36%)
46–65 years	103 (41%)
≥66 years	30 (12%)
Nationality	
Kuwaiti	206 (81%)
Non-Kuwaiti	47 (19%)
Gender	
Male	115 (45%)
Female	138 (55%)

Values are frequencies and percentages.

### 4.2 Principal component analysis

**Figure 1:**
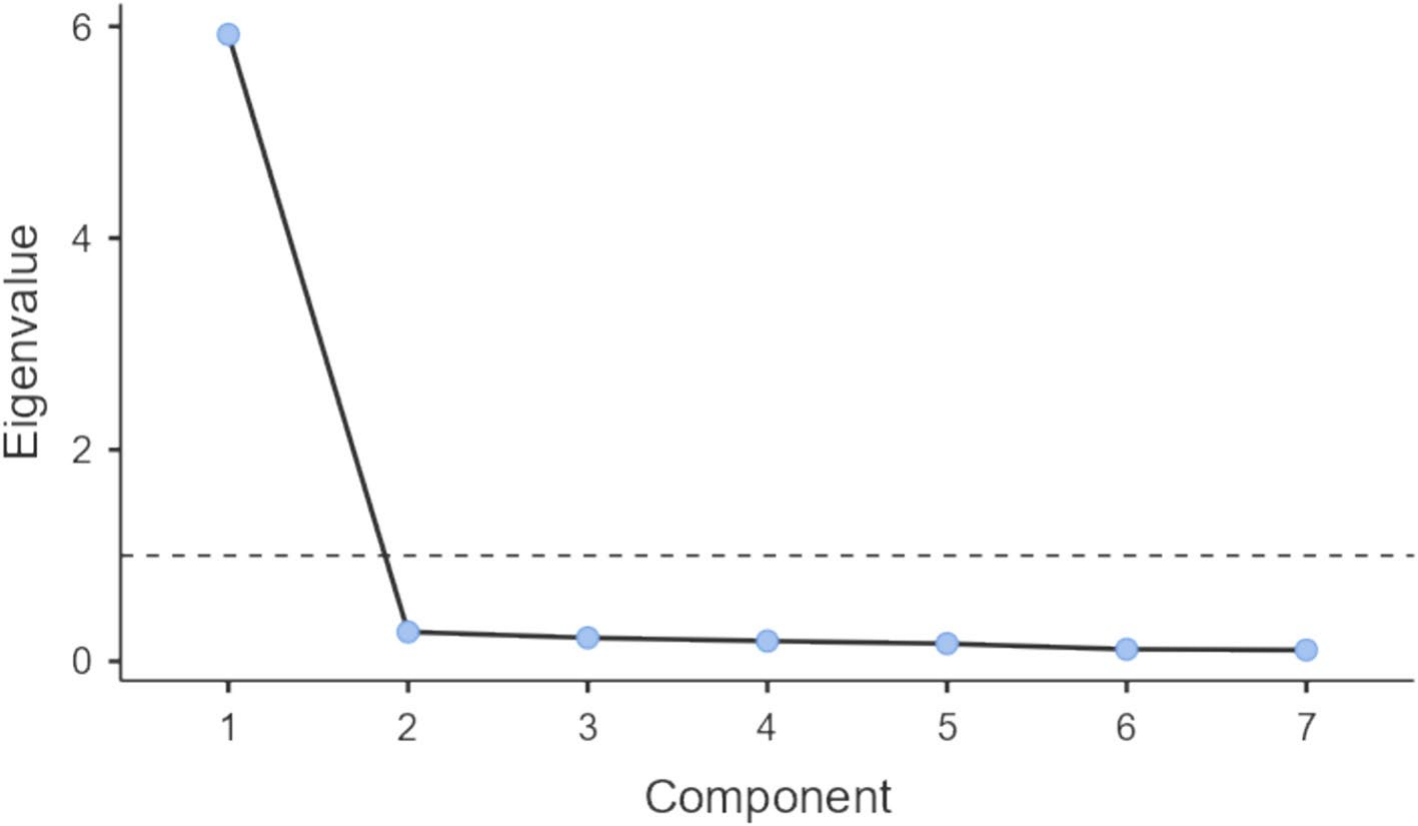
Scree plot.

Prior to PCA, the correlation matrix was evaluated for factorability. The KMO measure of sampling adequacy was 0.949, indicating meritorious adequacy (18). Bartlett’s test of sphericity was statistically significant (*χ*^2^ = 2,210, *df* = 21, *p* < 0.001), confirming that the correlation matrix was significantly different from an identity matrix and suitable for factor analysis. PCA yielded a single component with an eigenvalue greater than one. The scree plot (Figure 1) demonstrated a clear elbow after the first component, with all subsequent components contributing minimal additional variance, providing strong support for a unidimensional structure.

### 4.3 Scale reliability

**Figure 2:**
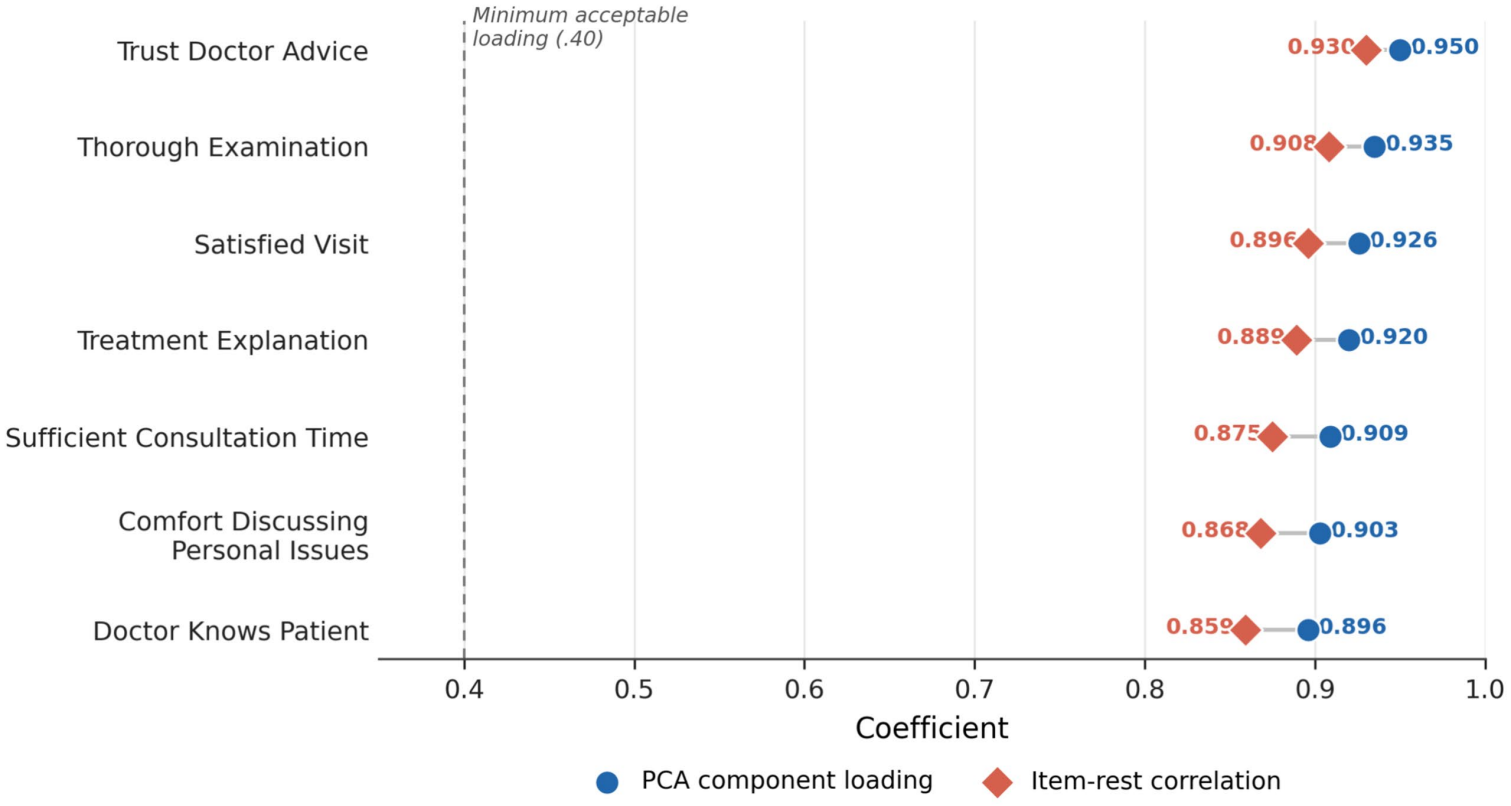
Principal component loadings and item-rest correlations for the seven satisfaction items. Items are ordered by descending component loading. Loadings are derived from the single-component principal component analysis solution; item-rest correlations represent the correlation between each item and the sum of the remaining items. All loadings (0.896–0.950) and item-rest correlations (0.859–0.930) substantially exceed the conventional minimum acceptable threshold of 0.40 (dashed line), supporting a strongly unidimensional scale.

The seven-item Patient Satisfaction Scale demonstrated excellent internal consistency (Cronbach’s *α* = 0.969; Table 2). Item-rest correlations were uniformly high, ranging from 0.859 (Doctor Knows Patient) to 0.930 (Trust Doctor Advice; Figure 2). Alpha-if-item-deleted values remained stable at 0.961–0.966, indicating that no single item meaningfully detracted from or inflated the scale’s reliability.

**Table 2:** Item-level reliability statistics for overall patient satisfaction.

Item	Mean	SD	Item-rest correlation	*α* if item dropped	Loading
Satisfied Visit	4.73	0.590	0.896	0.964	0.926
Thorough Examination	4.74	0.588	0.908	0.963	0.935
Treatment Explanation	4.72	0.603	0.889	0.964	0.920
Trust Doctor Advice	4.71	0.597	0.930	0.961	0.950
Doctor Knows Patient	4.70	0.640	0.859	0.966	0.896
Comfort Discussing Personal Issues	4.67	0.655	0.868	0.966	0.903
Sufficient Consultation Time	4.76	0.592	0.875	0.965	0.909

Overall Cronbach’s *α* = 0.969, Items were rated on a five-point Likert scale (1 = Very Dissatisfied to 5 = Very Satisfied). SD, standard deviation. Item-rest *r* = correlation between each item and the remaining scale total. *α* if deleted = Cronbach’s *α* recalculated after removing the specified item. Loading = principal component loading from the single-factor PCA solution. KMO = 0.949; Bartlett’s *χ*^2^ = 2,210, *df* = 21, *p* < 0.001.

### 4.4 Descriptive statistics and response distributions

Item-level means ranged from 4.67 (SD = 0.655; Comfort Discussing Personal Issues) to 4.76 (SD = 0.592; Sufficient Consultation Time). The composite Overall Satisfaction score had a mean of 4.72 (SD = 0.560; Table 3).

**Table 3:** Descriptive statistics for the composite Overall Satisfaction score.

	Mean	SD	Skewness	SE	Kurtosis	SE
Overall Satisfaction	4.72	0.560	−3.59	0.153	18.0	0.305

SD, standard deviation; SE, standard error. The composite Overall Satisfaction score is the mean of the seven items. Negative skewness reflects clustering of responses toward the high-satisfaction end of the scale. Elevated kurtosis is consistent with a pronounced ceiling effect. These distributional characteristics informed the selection of non-parametric tests for group comparisons.

The distribution of the composite score was markedly negatively skewed (skewness = −3.59, SE = 0.153) and leptokurtic (kurtosis = 18.0, SE = 0.305), indicating substantial departure from normality and strong clustering of responses at the highest satisfaction levels.

**Figure 3:**
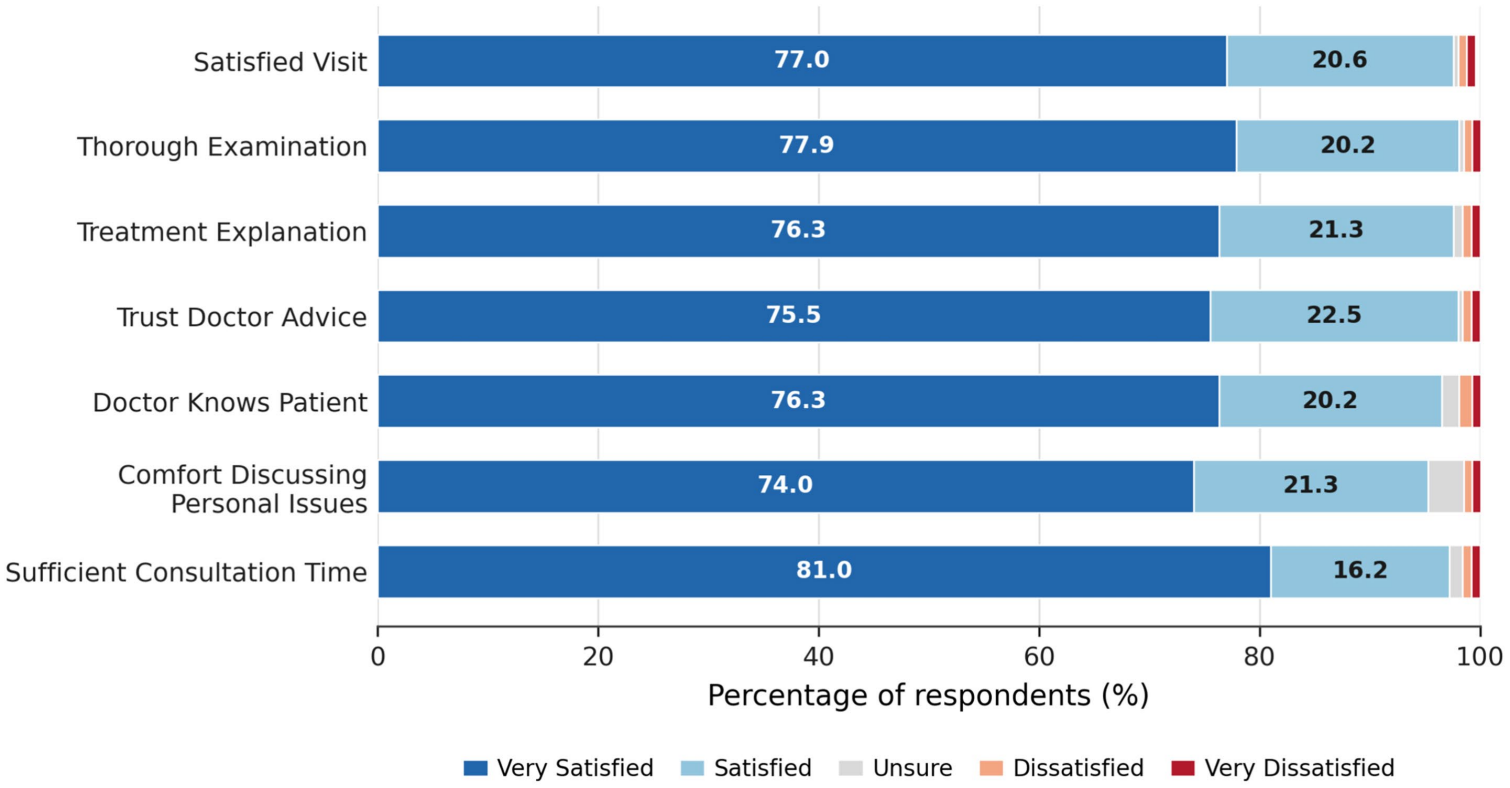
Distribution of patient responses across the five-point satisfaction scale for each of the seven items. Values are percentages (*N* = 253). Bars are scaled to 100%; numeric labels are shown for the Very Satisfied and Satisfied categories. The dominance of the Very Satisfied category (74.0–81.0% across all items) illustrates the pronounced ceiling effect, with Dissatisfied and Very Dissatisfied responses jointly accounting for no more than 2.0% of responses per item.

Response distributions for individual items are presented in Table 4, Figure 3. Across all seven items, 74.0–81.0% of respondents selected the highest category (Very Satisfied), indicating a pronounced ceiling effect. Satisfied responses accounted for an additional 16.2–22.5% per item. Dissatisfied and Very Dissatisfied responses collectively accounted for no more than 2.0% of responses for any single item.

**Table 4:** Response distribution for patient–doctor relationship items (*N* = 253).

Variable	Very Satisfied	Satisfied	Unsure	Dissatisfied	Very Dissatisfied
	*n* (%)	*n* (%)	*n* (%)	*n* (%)	*n* (%)
Satisfied Visit	196 (77.0)	52 (20.6)	1 (0.4)	2 (0.8)	2 (0.8)
Thorough Examination	197 (77.9)	51 (20.2)	1 (0.4)	2 (0.8)	2 (0.8)
Treatment Explanation	193 (76.3)	54 (21.3)	2 (0.8)	2 (0.8)	2 (0.8)
Trust Doctor Advice	191 (75.5)	57 (22.5)	1 (0.4)	2 (0.8)	2 (0.8)
Doctor Knows Patient	193 (76.3)	51 (20.2)	4 (1.6)	3 (1.2)	2 (0.8)
Comfort Discussing Personal Issues	187 (74.0)	54 (21.3)	8 (3.2)	2 (0.8)	2 (0.8)
Sufficient Consultation Time	205 (81.0)	41 (16.2)	3 (1.2)	2 (0.8)	2 (0.8)

Values are *n* (%). Percentages may not total 100 due to rounding. Across all items, 74.0–81.0% of respondents selected the highest satisfaction category (Very Satisfied), indicating a pronounced ceiling effect. Dissatisfied and Very Dissatisfied responses collectively accounted for no more than 2.0% of responses for any single item.

### 4.5 Kruskal–Wallis tests and *post-hoc* comparisons

Given evidence of non-normality in the composite satisfaction score, Kruskal–Wallis tests were used to compare Overall Satisfaction across demographic subgroups (Table 5).

**Table 5:** Kruskal–Wallis comparisons of Overall Satisfaction by demographic characteristic.

Characteristic	*χ* ^2^	*df*	*p*	ε^2^	Pairwise comparisons
Age	24.60	4	<0.001	0.098	46–65 years > 25–45 years (*p* < 0.001); ≥66 years >25–45 years (*p* = 0.029)
Nationality	0.12	1	0.732	<0.001	—
Gender	0.01	1	0.913	<0.001	—

*N* = 253. The composite Overall Satisfaction score was compared across subgroups using the Kruskal–Wallis test, which was selected because the score was markedly non-normally distributed. *χ*^2^ = Kruskal–Wallis test statistic; *df* = degrees of freedom; *ε*^2^ = epsilon-squared effect size (0.01 = small, 0.06 = medium, 0.14 = large). Significant pairwise differences were identified using Dunn’s test with Bonferroni correction; dashes (—) denote that no pairwise comparisons were performed because the overall test was non-significant. Achieved statistical power for the age-group comparison was 0.94 (*α* = 0.05).

A statistically significant difference in Overall Satisfaction was observed across age groups (*χ*^2^ = 24.60, *df* = 4, *p* < 0.001, *ε*^2^ = 0.098), indicating a small-to-moderate effect. Dunn’s *post-hoc* tests with Bonferroni correction revealed that participants aged 46–65 years reported significantly higher satisfaction than those aged 25–45 years (*p* < 0.001), and participants aged ≥66 years reported significantly higher satisfaction than those aged 25–45 years (*p* = 0.029). No other pairwise comparisons reached statistical significance.

No significant differences in Overall Satisfaction were observed by nationality (*χ*^2^ = 0.12, *df* = 1, *p* = 0.732, *ε*^2^ < 0.001) or gender (*χ*^2^ = 0.01, *df* = 1, *p* = 0.913, *ε*^2^ < 0.001). A *post-hoc* power analysis indicated that, for the age-group comparison (*ε*^2^ = 0.098, five groups, *α* = 0.05), achieved power at *n* = 253 was 0.94, exceeding the conventional 0.80 threshold.

## 5 Discussion

This cross-sectional study described patient satisfaction with a family medicine appointment clinic at the Nassem PHCC in Kuwait. Patients reported high Overall Satisfaction, a unidimensional scale structure was confirmed, and significant age-group differences in satisfaction were observed, whereas nationality and gender were unrelated to satisfaction. Collectively, these findings offer preliminary observational evidence of positive patient perceptions of structured, continuity-based family medicine care within Kuwait’s primary health system (11, 23, 24).

The seven-item scale demonstrated excellent internal consistency (Cronbach’s *α* = 0.969), substantially exceeding the 0.70 threshold for research instruments and the 0.90 threshold for clinical-grade measures (25). Item-rest correlations (0.859–0.930) and stable alpha-if-item-deleted values (0.961–0.966) confirm that all items contributed independently to the composite score. PCA yielded a single component with a KMO of 0.949, a statistically significant Bartlett’s test, and component loadings uniformly exceeding 0.89, providing strong evidence of unidimensionality consistent with Baker’s (16) original framework and prior Arabic-adapted applications in Gulf primary care (4). Within the expectancy–disconfirmation framework, the uniformly high ratings across all items—spanning thoroughness of examination, treatment explanation, trust in the physician, and adequacy of consultation time—indicate that the structured appointment model met or exceeded patient expectations on the interpersonal dimensions of care (26). This aligns with systematic-review evidence identifying the provider–patient relationship as the most consistent determinant of patient satisfaction, and the convergence of all items on a single factor suggests that patients appraised their care holistically through this relational lens (26).

A pronounced ceiling effect was documented, with 74–81% of respondents selecting the maximum satisfaction category and an extreme composite score distribution (skewness = −3.59; kurtosis = 18.0). While ceiling effects in satisfaction surveys can partly reflect genuinely high care quality, the distribution observed here is considerably more extreme than that typically reported for patient-reported outcome measures, indicating that the instrument captures meaningful variation only within a narrow band near the top of the scale and cannot sensitively differentiate between satisfied and highly satisfied patients (27, 28). The ceiling effect was likely amplified by social desirability bias: questionnaires were administered by treating physicians immediately after consultation, which may have generated implicit social pressure to respond favourably—particularly in a cultural context where overt criticism of healthcare providers may be uncomfortable (27, 29). The extremely low proportion of dissatisfied responses (no more than 2.0% per item) is consistent with this interpretation and warrants cautious interpretation of the reported satisfaction levels. Future studies should consider independently administered, anonymous surveys, time-delayed data collection, or supplementary qualitative methods to elicit experiences that patients may be reluctant to report on structured questionnaires.

The high satisfaction observed here is broadly consistent with findings from structured primary care settings across the GCC. A scoping review by Alhenaidi et al. (6) reported consistently high satisfaction across Saudi Arabia, UAE, and Qatar, with physician–patient communication and consultation time identified as the most influential predictors—a pattern mirrored in the present sample, where Sufficient Consultation Time attracted the highest proportion of Very Satisfied responses (81.0%). Studies from Saudi Arabia have similarly documented positive patient perceptions of appointment-based primary care, particularly regarding physician availability and reduced waiting times, while within Kuwait, Badawi et al. (13) documented fragmented chronic disease management in walk-in PHCCs—providing contextual contrast with the continuity-based model evaluated here. Internationally, systematic reviews demonstrate that longitudinal relationships with a named clinician are associated with lower hospitalisation rates, reduced mortality, and higher patient satisfaction (3, 30, 31). The same-physician follow-up policy and 20-min consultation allocation at Nassem PHCC are structural features aligned with these continuity principles. While causal attribution is not possible from a cross-sectional study, the pattern of findings—particularly high ratings for consultation thoroughness and time adequacy—is congruent with the broader evidence base on continuity-based primary care, and adds preliminary observational support to a small but growing body of literature on the patient acceptability of appointment-based family medicine within Kuwait’s PHCC system.

The significant age effect (Kruskal–Wallis *χ*^2^ = 24.60, *p* < 0.001, *ε*^2^ = 0.098) is consistent with the well-replicated finding that older patients report higher primary care satisfaction than younger adults (32, 33). *Post-hoc* comparisons showed that patients aged 46–65 years and those aged 66 years and older were both significantly more satisfied than patients aged 25–45 years. Proposed explanations include differences in healthcare expectations, familiarity with care systems, and preferences for continuity: older adults tend to value the personalised, relationship-oriented characteristics of family medicine, whereas younger adults may hold higher expectations regarding convenience, digital access, and scheduling flexibility (3, 6, 23). This age disparity has practical implications for service improvement: targeted initiatives for patients aged 25–45 years—including improved digital appointment booking, communication strategies adapted to younger preferences, and clearer orientation to the family medicine model—may help to reduce satisfaction gaps.

No significant differences in satisfaction were observed by gender (*ε*^2^ < 0.001) or nationality (*ε*^2^ < 0.001), with negligible effect sizes suggesting minimal practical relevance of these variables in this sample. This is consistent with GCC studies finding no gender differential in primary care satisfaction once structural factors are held constant (6). However, given the predominantly Kuwaiti (81.4%) and female (54.5%) sample composition, statistical power to detect subgroup effects was limited, and these null results should be interpreted with caution. More broadly, sociodemographic predictors of satisfaction vary inconsistently across the international literature and are best treated as potential confounders rather than stable determinants (34).

From a policy and implementation perspective, the Nassem PHCC appointment system integrates electronic and manual registration, aligning with broader GCC trends in digital health transformation (23). Structured scheduling supports continuity of care, reduces physician workload uncertainty, and may improve consultation quality through predictable patient flow—factors that patients are likely to perceive positively (35). Under Kuwait Vision 2035, broader implementation of family medicine in primary care has been identified as a strategic priority, and the present findings may provide useful preliminary information for policymakers in this context. However, given the cross-sectional design, convenience sampling, and absence of a comparator group, multi-centre and comparative studies are required before these findings can substantively inform large-scale implementation decisions.

## 6 Strengths and limitations

### 6.1 Strengths

This study has several notable methodological and analytical strengths. It is among the first studies in Kuwait to evaluate patient satisfaction with a family medicine appointment clinic using a formally validated instrument with comprehensive psychometric assessment, including PCA, KMO, Bartlett’s test, and item-level reliability analysis. Non-parametric statistical methods (Kruskal–Wallis tests, Dunn *post-hoc* comparisons with Bonferroni correction, epsilon-squared effect sizes) were fully justified by the distributional properties of the data. Ceiling effects were explicitly quantified. A *post-hoc* power analysis confirmed adequate power (0.94) for the primary age-group comparison despite the achieved sample size falling short of the *a priori* target.

### 6.2 Limitations

Several limitations require careful consideration. First and most importantly, the cross-sectional design precludes causal inference. The absence of a comparator group, baseline data, or pre-implementation assessment means it is not possible to determine whether observed satisfaction levels are attributable to the family medicine model, the appointment system, physician characteristics, or other unmeasured factors. All interpretations should be read as observational and associational.

Second, social desirability bias is a material concern. Questionnaires were administered immediately after consultation and distributed by the treating physician. Patients may have felt implicit social pressure to respond favourably, particularly in a cultural context where criticism of a healthcare provider may be uncomfortable. Social desirability effects of this nature can inflate satisfaction ratings and contribute to ceiling effects, as documented in healthcare satisfaction research (10, 27). Future studies should consider anonymous administration independent of the treating clinician.

Third, convenience sampling from a single PHCC introduces the risk of selection bias. Patients who declined participation, attended on non-recruitment days, or experienced less satisfactory consultations may be systematically underrepresented. Single-centre recruitment in one urban district limits external validity: findings may not generalise to other PHCCs, governorates, or patient populations with different demographic or clinical profiles. Multi-centre studies across diverse Kuwaiti PHCCs are needed to establish more widely applicable conclusions.

Fourth, only three demographic variables were collected, precluding multivariable regression or adjustment for potential confounders such as educational attainment, chronic disease burden, visit frequency, waiting time, or health literacy. This limits the explanatory depth of the findings.

Fifth, the pronounced ceiling effect substantially reduces the discriminant validity of the scale in this high-satisfaction population, limiting its utility for detecting incremental improvements in care quality. Instruments with wider response ranges or supplementary qualitative methods are recommended for future studies.

## 7 Conclusion

Patients attending the family medicine appointment clinic at the Nassem PHCC in Kuwait reported consistently high satisfaction with the doctor–patient relationship across every aspect assessed, and the questionnaire performed reliably and coherently in this setting. Ratings were so uniformly favourable that the instrument had limited ability to distinguish finer differences in patient experience. Older patients reported greater satisfaction than younger adults, whereas satisfaction did not differ by gender or nationality. Taken together, these findings offer preliminary, observational evidence of positive patient perceptions of structured, continuity-based family medicine care within Kuwait’s primary health system. Because the study drew on a convenience sample at a single centre, with questionnaires completed immediately after the consultation, the results may overstate satisfaction and cannot establish cause and effect. Longitudinal, multi-centre, and comparative studies using more discriminating measures are needed to strengthen the evidence base for wider implementation of the family medicine model in Kuwait and the broader Gulf Cooperation Council region.

## Data Availability

The raw data supporting the conclusions of this article will be made available by the authors, without undue reservation.
